# The Book World of Medicine and Science

**Published:** 1904-02-20

**Authors:** 


### THE BOOK WORLD OF MEDICINE AND SCIENCE.

MORALS: A TREATISE ON THE PSYCHO-SOCIOLOGICAL BASES OF ETHICS. By Professor G. L. DUPRAT. Translated (from the French) by W. J. Greenstreet, M.A., F.R.A.S. (The Walter Scott Publishing Co., Ltd. 1903. Pp xv., 382. Price 6s.)

MORALS in the melting-pot is the impression left upon the old-fashioned reader in turning the pages of this treatise. Not even in ethics is there finality. Aristotle's teaching on the virtues and vices has been authoritative for a long time, and has been practically adopted by the religious world. "While the world lasts," says Newman, "will Aristotle's doctrine on these matters last, for he is the oracle of nature and of truth." But we have become physiological in our methods, and above all evolutional in our point of view. There are mental and moral aberrations from the normal, leading to insanity and crime, the scientific study of which has stirred the whole question of responsibility, resentment, and punishment. Hence an extensive crop of writings, in which the Italian school of Lombroso has taken a leading part. It is of this new, comprehensive, scientific basis of morals that the treatise of Professor Duprat on "La Morale" gives a systematic account, sub-divided and paragraphed like a handbook, and furnished with an immense number of references to the contemporary literature. The subject, he points out, has been largely in the hands of medical men, who are constantly brought face to face with its problems in one form, just as the police magistrates and the criminal lawyers are confronted with them in another. He says in his preface: "Whoever wishes to lay down rules for the guidance of the conduct of his fellow-creatures must be a *savant* before he is a moralist. . . . The doctor-philosopher of to-day who, following men like Charcot, Ribot, and Janet, has introduced into psychology an entirely new spirit, now applies to the moral life the really scientific knowledge he has acquired in his clinical work, in the laboratory, in the hospital, and in the asylum; he thus welds together two links in a chain—the study of nervous or mental diseases, and the struggle against social diseases, *i.e.*, against immorality" Moralists with strong prejudices, such as good old Samuel Johnson, have always been alive to the dangers of reconsidering the bases of morals, in theory at least. Johnson was ready enough to pronounce, "The man's a scamp, and there's an end on't;" but in his "Life of Savage" he was very tender to an individual of the genus scamp, just because he liked him. The scientific reconsideration of these matters has the warrant of Scripture itself. What can be mcre fixed than the Decalogue? In the Second Commandment we find the principle of "visiting the sins of the fathers upon the children unto the third and fourth generation of them that hate me." And yet this law of heredity is challenged in the prophecies of Jeremiah, and is made the subject of a whole chapter of vehement remonstrance in the Book of Ezekiel: "Ye shall not have occasion any more to use this proverb in Israel, The fathers have eaten sour grapes, and the children's teeth are set on edge." This refusal to acquiesce in social respectability or the reverse had a scientific basis then, just as much as the sociological doctrine of morals has now. It has been always among the Jews that the phenomenon of alternation of generations has been most obvious, that strange reaction in the lofty idealism of the son from the lower aims of the father, which Freytag has made the theme of his story "Soll und Haben" (Debit and Credit). The older view of heredity was too simple for the facts, so that the writer of the 18th chapter of Ezekiel feels himself on sure ground when he traverses it with another divine oracle, "All souls are mine." Not different is the point of view of those who appeal to scientific observation now, although they have a bewildering complexity of facts to deal with. The Contemporary Science Series has already included monographs on several special subjects in practical sociology. This treatise by M. Duprat should serve as a general philosophical introduction, as it is psychological in its method, and touches the practical subjects on every page. It is not at all aggressive in manner, but positive, and therefore to be welcomed.

DRUGS, THEIR PRODUCTION, PREPARATION, AND PROPERTIES. By H. WIPPELL GADD, F.C.S., M.P.S. Crown 8vo. Pp. xii–180. (London: Baillière, Tindall and Cox. Price 5s. net.)

THIS small book may be highly commended to the junior student of materia medica. It hits the happy mean between the bald, scrappy "cram" manual on the one hand, and the exhaustive treatise suitable only for experts and as a work of reference on the other. The author's aim is to give a concse account of the principal vegetable, animal, and chemical substances used in medicine. In doing this he displays an admirable sense of proportion and a happy ingenuity by which the severity of his theme is pleasantly relieved. The majority of textbooks dealing with this subject fail to realise that the student needs the exposition of principles and not details of mere manufacturing interest. The latter have their place, but this is not in a manual intended as an introduction to the study of drugs. Mr. Gadd has fully grasped the position of the junior student, and consequently his book is a real instrument of education. It provides a secure basis on which an exact and comprehensive knowledge of principles may be grounded. We would venture to suggest that the section dealing with pharmaceutical processes might have been extended with advantage, and that the solubility or otherwise of the various mineral salts used in medicine should in every instance be stated. These points may perhaps receive attention in a second edition. That this will soon be demanded we cannot doubt, for of the numerous manuals which deal with materia medica we know of none which so pleasantly and successfully presents to the reader the essential facts and principles of this important subject.

STREET'S NEWSPAPER DIRECTORY, 1904. (London: G. Street and Co., 8 Serle Street, W.C.)

THIS is a work of much utility to all who have to do with newspapers, containing as it does a complete alphabetical list of newspapers and periodicals with their prices, days of publication, addresses, etc. In addition there are classified lists according to the special trade or interests served and of the towns in which they are published. To make all quite clear and easy some maps of the British Isles are given, and much useful information as to population, etc., of Provincial towns is also supplied.

